# Supplementary material for: Gradient boosted decision trees reveal nuances of auditory discrimination behavior
Source: PLoS Comput Biol. 2024 Apr 16;20(4):e1011985. doi: 10.1371/journal.pcbi.1011985 (PMC11051626; doi:10.1371/journal.pcbi.1011985)
Supplement: S1 Table — (PDF) [file pcbi.1011985.s008.pdf]

S1 Table

Within-group factor	SS	Degrees of freedom (numerator)	Degrees of freedom (denominator)	MS	F-value	Uncorrected p-value	GG corrected p-value	Generalized eta-squared	GG epsilon factor
roving_type	0.0002851	2	8	0.00014255	0.1673	0.8488	0.8339	0.0037	0.9247
talker	0.050970082	1	4	0.050970082	12.0173	0.0257	0.0257	0.3979	1
roving_type * talker	0.0215	2	8	0.0108	16.3772	0.0015	0.0099	0.2181	0.5936

S1 Table: Repeated-measures ANOVA for the hit statistic with roving type and talker as factors.
