# Supplementary material for: Gradient boosted decision trees reveal nuances of auditory discrimination behavior
Source: PLoS Comput Biol. 2024 Apr 16;20(4):e1011985. doi: 10.1371/journal.pcbi.1011985 (PMC11051626; doi:10.1371/journal.pcbi.1011985)
Supplement: S2 Table — (PDF) [file pcbi.1011985.s009.pdf]

S2 Table

A	B	mean(A)	mean(B)	diff	se	T	p-tukey	hedges	talker
control	inter	0.8280	0.8823	-0.0542	0.0390	-1.3916	0.3756	-0.7149	Female
control	intra	0.8280	0.8738	-0.0458	0.0390	-1.1752	0.4893	-0.6126	Female
inter	intra	0.8823	0.8738	0.0084	0.0390	0.2164	0.9746	0.1648	Female
control	inter	0.8201	0.7508	0.0693	0.0324	2.1367	0.1239	1.0451	Male
control	intra	0.8201	0.7659	0.0542	0.0324	1.6725	0.2551	1.3949	Male
inter	intra	0.7508	0.7659	-0.0151	0.0324	-0.4642	0.8891	-0.2455	Male

S2 Table: Pairwise Tukey HSD posthoc test statistics for the hit statistic comparing the roving types for each talker type.
