# Supplementary material for: Gradient boosted decision trees reveal nuances of auditory discrimination behavior
Source: PLoS Comput Biol. 2024 Apr 16;20(4):e1011985. doi: 10.1371/journal.pcbi.1011985 (PMC11051626; doi:10.1371/journal.pcbi.1011985)
Supplement: S3 Table — (PDF) [file pcbi.1011985.s010.pdf]

S3 Table

Within-group factor	SS	Degrees of freedom (numerator)	Degrees of freedom (denominator)	MS	F-value	Uncorrected p-value	GG corrected p-value	Generalized eta-squared	GG epsilon factor
roving_type	0.0347	2	8	0.0174	32.541	0.0001436	0.0011539	0.3932	0.6985
talker	0.0057	1	4	0.0057	7.7165	0.04993	0.04993	0.0961	1
roving_type * talker	0.0087	2	8	0.0043	16.3283	0.0014991	0.0082412	0.1391	0.6338

S3 Table: Repeated-measures ANOVA for the false alarm statistic with roving type and talker as factors
