# Supplementary material for: Gradient boosted decision trees reveal nuances of auditory discrimination behavior
Source: PLoS Comput Biol. 2024 Apr 16;20(4):e1011985. doi: 10.1371/journal.pcbi.1011985 (PMC11051626; doi:10.1371/journal.pcbi.1011985)
Supplement: S4 Table — (PDF) [file pcbi.1011985.s011.pdf]

S4 Table

A	B	mean(A)	mean(B)	diff	se	T	p-tukey	hedges	talker
control	inter	0.1213	0.2395	-0.1181	0.0288	-4.0986	0.0039	-2.2667	Female
control	intra	0.1213	0.2069	-0.0855	0.0288	-2.9677	0.0294	-2.2529	Female
inter	intra	0.2395	0.2069	0.0326	0.0288	1.1310	0.5143	0.5526	Female
control	inter	0.1969	0.2445	-0.0476	0.0309	-1.5418	0.3072	-0.8621	Male
control	intra	0.1969	0.2089	-0.0120	0.0309	-0.3884	0.9207	-0.2324	Male
inter	intra	0.2445	0.2089	0.0356	0.0309	1.1533	0.5016	0.6446	Male

S4 Table: Pairwise Tukey HSD posthoc test statistics for the false alarm statistic comparing the roving types for each talker type.
