# Supplementary material for: Gradient boosted decision trees reveal nuances of auditory discrimination behavior
Source: PLoS Comput Biol. 2024 Apr 16;20(4):e1011985. doi: 10.1371/journal.pcbi.1011985 (PMC11051626; doi:10.1371/journal.pcbi.1011985)
Supplement: S5 Table — (PDF) [file pcbi.1011985.s012.pdf]

S5 Table

Within-group factor	SS	Degrees of freedom (numerator)	Degrees of freedom (denominator)	MS	F-value	Uncorrected p-value	GG corrected p-value	Generalized eta-squared	GG epsilon factor
roving_type	0.4941	2	8	0.2470	19.0512	0.0009067	0.0016395	0.2171	0.8836
talker	1.5566	1	4	1.5566	13.3321	0.0217	0.0217	0.4662	1
roving_type * talker	0.0379	2	8	0.0190	1.4193	0.2968	0.3006	0.0208	0.5931

S5 Table: Repeated-measures ANOVA for the d' statistic with roving type and talker as factors
