# Supplementary material for: Gradient boosted decision trees reveal nuances of auditory discrimination behavior
Source: PLoS Comput Biol. 2024 Apr 16;20(4):e1011985. doi: 10.1371/journal.pcbi.1011985 (PMC11051626; doi:10.1371/journal.pcbi.1011985)
Supplement: S6 Table — (PDF) [file pcbi.1011985.s013.pdf]

S6 Table

A	B	mean(A)	mean(B)	diff	se	T	p-tukey	hedges
control	inter	1.9759	1.6675	0.3084	0.1582	1.9500	0.1443	0.8036
control	intra	1.9759	1.7690	0.2070	0.1582	1.3086	0.4028	0.5891
inter	intra	1.6675	1.7690	-0.1014	0.1582	-0.6414	0.7987	-0.2727

S6 Table: Pairwise Tukey HSD posthoc test statistics for the d' statistic comparing the roving type.
