# Supplementary material for: Gradient boosted decision trees reveal nuances of auditory discrimination behavior
Source: PLoS Comput Biol. 2024 Apr 16;20(4):e1011985. doi: 10.1371/journal.pcbi.1011985 (PMC11051626; doi:10.1371/journal.pcbi.1011985)
Supplement: S7 Table — (PDF) [file pcbi.1011985.s014.pdf]

S7 Table

	Coefficients	p-values	Std Error	Reference Var.
Intercept	0.089651781	0.793205158	0.348559363	NA
talker[T.Female]	-1.433112675	6.82×10^{-10}	0.21701492	Male
side[T.Right]	0.872739206	4.45×10^{-15}	0.106516924	Left
precur_and_targ_same[T.1.0]	-0.091146086	0.552594672	0.129515117	0
pastcorrectresp[T.1]	0.107175605	0.6718157	0.215592582	0
pastcatchtrial[T.1]	0.104160869	0.423572557	0.121099972	109 Hz
pitchoftarg[T.124 Hz]	-0.342035193	0.069402262	0.163079644	109 Hz
pitchoftarg[T.144 Hz]	-0.106131185	0.509131474	0.178582717	109 Hz
pitchoftarg[T.191 Hz]	0.639913761	0.027903865	0.273003545	109 Hz
pitchoftarg[T.251 Hz]	-0.168676515	0.509841226	0.301580641	NA
targTimes	-0.031639904	0.448994716	0.041144214	NA

S7 Table: Average coefficients of the main fixed effects of the miss/correct generalized linear mixed effects response model.
