# Supplementary material for: Gradient boosted decision trees reveal nuances of auditory discrimination behavior
Source: PLoS Comput Biol. 2024 Apr 16;20(4):e1011985. doi: 10.1371/journal.pcbi.1011985 (PMC11051626; doi:10.1371/journal.pcbi.1011985)
Supplement: S8 Table — (PDF) [file pcbi.1011985.s015.pdf]

S8 Table

Variable	Value
F1702	0.369810812
F1815	-0.454596914
F1803	-0.486838133
F2002	-0.050446788
F2105	0.622900046

S8 Table: Average coefficients of the random effects for the miss/correct generalized linear mixed effects response model.
