# Supplementary material for: Gradient boosted decision trees reveal nuances of auditory discrimination behavior
Source: PLoS Comput Biol. 2024 Apr 16;20(4):e1011985. doi: 10.1371/journal.pcbi.1011985 (PMC11051626; doi:10.1371/journal.pcbi.1011985)
Supplement: S9 Table — 1 indicates yes, 0 indicates no. (PDF) [file pcbi.1011985.s016.pdf]

S9 Table

	Coefficients	p-values	Std Error	Reference Var.
Intercept	0.732556885	0.000487712	0.178742303	NA
talker[T.Female]	-0.12858336	0.160216905	0.082143402	Male
audio_side[T.Right]	-0.059413078	0.27476522	0.047805183	Left
intra_trial_F0_roving[T.1]	-0.013491969	0.57768937	0.050284619	0
past_response_correct[T.1]	-0.483403258	1.68×10^{-6}	0.089589251	0
past_trial_was_catch[T.1]	0.056375781	0.331617868	c 0.054798595	0
F0[T.124 Hz]	-0.126915622	0.119056884	0.076903337	109 Hz
F0[T.144 Hz]	-0.128452028	0.12414751	0.075913927	109 Hz
F0[T.191 Hz]	-0.69952142	2.44×10^{-7}	0.119227149	109 Hz
F0[T.251 Hz]	-0.144339973	0.296305056	0.116080756	109 Hz
time_since_trial_start	0.071926936	0.003653682	0.024114444	NA
trial_number	-0.175637965	4.08×10^{-10}	0.025124411	NA

S9 Table: Average fixed effect coefficients for false alarm generalized linear mixed-effects model. 1 indicates yes, 0 indicates no.
