# Supplementary material for: Gradient boosted decision trees reveal nuances of auditory discrimination behavior
Source: PLoS Comput Biol. 2024 Apr 16;20(4):e1011985. doi: 10.1371/journal.pcbi.1011985 (PMC11051626; doi:10.1371/journal.pcbi.1011985)
Supplement: S10 Table — (PDF) [file pcbi.1011985.s017.pdf]

S10 Table

Variable	Value
F1702	0.16169911
F1815	0.058062191
F1803	0.183989004
F2002	0.104246127
F2105	-0.507602176

Table S10: Average random effect coefficients for the false alarm generalized linear mixed-effects model.
