# Supplementary material for: Gradient boosted decision trees reveal nuances of auditory discrimination behavior
Source: PLoS Comput Biol. 2024 Apr 16;20(4):e1011985. doi: 10.1371/journal.pcbi.1011985 (PMC11051626; doi:10.1371/journal.pcbi.1011985)
Supplement: S11 Table — 1 indicates yes, and 0 indicates no. (PDF) [file pcbi.1011985.s018.pdf]

S11 Table

	coefficients	p_values	std_dev.	reference var.
Intercept	0.7082	1.43E-41	0.0507	NA
target_F0[T.124 Hz]	-0.0936	4.40E-05	0.0214	109 Hz
target_F0[T.144 Hz]	0.0231	0.3965	0.0251	109 Hz
target_F0[T.191 Hz]	0.0271	0.4630	0.0333	109 Hz
target_F0[T.251 Hz]	-0.0241	0.4955	0.0345	109 Hz
past_trial_catch[T.1]	-0.0061	0.6117	0.0140	0
talker[T.Female]	-0.1509	5.63E-08	0.0250	Male
side_of_audio[T.Right]	0.0555	1.13E-05	0.0120	Left
precursor_equals_target_F0[T.1.0]	-0.0369	0.0063	0.0129	0
past_resp__correct[T.1]	0.0024	0.7772	0.0260	0
trial_no_	0.0004	0.0385	0.0002	NA
target_time	-0.0248	6.39E-07	0.0049	NA
Group Var	0.0841	0.1713	0.0615	NA

S11 Table: Average fixed effect coefficients for the reaction time linear mixed effects model for correct hit responses. 1 indicates yes, 0 indicates no.
