# Supplementary material for: Gradient boosted decision trees reveal nuances of auditory discrimination behavior
Source: PLoS Comput Biol. 2024 Apr 16;20(4):e1011985. doi: 10.1371/journal.pcbi.1011985 (PMC11051626; doi:10.1371/journal.pcbi.1011985)
Supplement: S12 Table — (PDF) [file pcbi.1011985.s019.pdf]

S12 Table

F1702	0.0803
F1815	0.0319
F1803	0.0536
F2002	-0.0414
F2105	-0.1244

S12 Table: Average random effect coefficients mixed effects model predicting reaction time for correct target trial responses.
