# Supplementary material for: Gradient boosted decision trees reveal nuances of auditory discrimination behavior
Source: PLoS Comput Biol. 2024 Apr 16;20(4):e1011985. doi: 10.1371/journal.pcbi.1011985 (PMC11051626; doi:10.1371/journal.pcbi.1011985)
Supplement: S13 Table — (PDF) [file pcbi.1011985.s020.pdf]

S13 Table

	coefficients	p_values	std_dev
Intercept	1.6652	0	0.0008
instruments	0.2501	0	0.0003
when_a	0.0933	0	0.0006
sailor	0.1327	0	0.0006
in_a_small	0.1194	0	0.0006
craft	0.1299	0	0.0006
faces	0.1296	0	0.0006
of_the_might	0.1227	0	0.0006
of_the_vast	0.1487	0	0.0006
atlantic	0.1278	0	0.0006
ocean	0.1085	0	0.0006
today	0.1052	0	0.0006
he_takes	0.1193	0	0.0006
the_same	0.1033	0	0.0006
risks	0.1250	0	0.0006
that_generations	0.1839	0	0.0006
took	0.0777	0	0.0006
before[0]	0.1184	0	0.0006
before[1]	0.1364	0	0.0005
him	0.0964	0	0.0006
but	0.1025	0	0.0006
in_contrast	0.1799	0	0.0006
them	0.1236	0	0.0006
he_can_meet	0.1377	0	0.0006
any	0.0855	0	0.0005
emergency	0.1552	0	0.0006
that_comes	0.1152	0	0.0006
his_way	0.1441	0	0.0006
confidence	0.1563	0	0.0006
that_stems	0.1326	0	0.0006
profound	0.1490	0	0.0006
trust	0.1159	0	0.0006
advance	0.1330	0	0.0006
of_science	0.1869	0	0.0006
boats	0.0887	0	0.0006
stronger	0.1242	0	0.0006
more_stable	0.1907	0	0.0006
protecting	0.1472	0	0.0006
against	0.1069	0	0.0006
and_du	0.1076	0	0.0006
exposure	0.1836	0	0.0006
tools_and	0.1371	0	0.0006
more_ah	0.0867	0	0.0006
accurate	0.1480	0	0.0006
the_more	0.0883	0	0.0005
reliable	0.1556	0	0.0006
helping_in	0.1260	0	0.0006
normal_weather	0.1227	0	0.0006
and_conditions	0.1737	0	0.0005
food	0.1154	0	0.0006
and_drink	0.1274	0	0.0006
of_better	0.1157	0	0.0006
researched	0.1577	0	0.0006
than_easier	0.1363	0	0.0006
to_cook	0.1101	0	0.0006
than_ever	0.1103	0	0.0006

S13 Table: Coefficients for the ordinary least squares (OLS) model predicting absolute reaction time based on word identity in a trial, female talker model.
