# Supplementary material for: Gradient boosted decision trees reveal nuances of auditory discrimination behavior
Source: PLoS Comput Biol. 2024 Apr 16;20(4):e1011985. doi: 10.1371/journal.pcbi.1011985 (PMC11051626; doi:10.1371/journal.pcbi.1011985)
Supplement: S14 Table — (PDF) [file pcbi.1011985.s021.pdf]

S14 Table

	coefficients	p_values	std_dev.
Intercept	1.6723	0	0.0008
instruments	0.1992	0	0.0003
when_a	0.0930	0	0.0005
sailor	0.1298	0	0.0005
in_a	0.0734	0	0.0005
small	0.1004	0	0.0005
craft	0.1115	0	0.0005
faces	0.1260	0	0.0005
the_might	0.1180	0	0.0005
of_the	0.0833	0	0.0005
vast	0.0957	0	0.0005
atlantic	0.1501	0	0.0005
ocean	0.1030	0	0.0005
today	0.1174	0	0.0005
he[0]	0.0484	0	0.0005
he[1]	0.0562	0	0.0005
takes	0.0942	0	0.0005
the_same	0.1188	0	0.0005
risks	0.1026	0	0.0005
that_generations	0.1774	0	0.0005
took	0.0531	0	0.0005
before_him	0.1408	0	0.0005
but	0.0788	0	0.0005
in_contrast	0.1495	0	0.0005
to_them	0.1053	0	0.0005
can_meet	0.0754	0	0.0005
any	0.0685	0	0.0005
emergency	0.1460	0	0.0005
that_comes	0.1233	0	0.0005
his_way	0.1336	0	0.0005
with_a	0.0620	0	0.0005
confidence	0.1287	0	0.0005
that_stems	0.1485	0	0.0005
_from	0.0839	0	0.0005
profound	0.1561	0	0.0005
trust	0.1045	0	0.0005
in_the	0.1107	0	0.0005
advances	0.1157	0	0.0005
of_science	0.1557	0	0.0005
boats	0.1129	0	0.0005
as_stronger	0.1488	0	0.0005
and_more[0]	0.1000	0	0.0005
and_more[1]	0.0863	0	0.0005
stable	0.1317	0	0.0005
protecting	0.1312	0	0.0005
against	0.1197	0	0.0005
undue	0.1309	0	0.0005
exposure	0.1753	0	0.0005
tools	0.1033	0	0.0005
accurate	0.1218	0	0.0005
reliable	0.1269	0	0.0005
helping	0.1206	0	0.0005
in_all	0.1209	0	0.0005
weather	0.0971	0	0.0005
a_n_d[0]	0.0586	0	0.0005
a_n_d[1]	0.0676	0	0.0005

S14 Table: Coefficients for the ordinary least squares (OLS) model predicting absolute reaction time based on word identity in a trial, male talker model.
