# Supplementary material for: Gradient boosted decision trees reveal nuances of auditory discrimination behavior
Source: PLoS Comput Biol. 2024 Apr 16;20(4):e1011985. doi: 10.1371/journal.pcbi.1011985 (PMC11051626; doi:10.1371/journal.pcbi.1011985)
Supplement: S15 Table — (PDF) [file pcbi.1011985.s022.pdf]

S15 Table

Ferret ID	F1702		F1815		F1803		F2002		F2105	
Talker	M	F	M	F	M	F	M	F	M	F
All trials	2834	2773	1758	1684	3060	3001	6381	3611	2026	2055
Catch trials	717	694	454	439	781	756	1583	918	504	519

S15 Table: Trial type numbers distributed by ferret ID and talker type (M = male talker, F= female talker)
