# Supplementary material for: Gradient boosted decision trees reveal nuances of auditory discrimination behavior
Source: PLoS Comput Biol. 2024 Apr 16;20(4):e1011985. doi: 10.1371/journal.pcbi.1011985 (PMC11051626; doi:10.1371/journal.pcbi.1011985)
Supplement: S16 Table — (PDF) [file pcbi.1011985.s023.pdf]

S16 Table

Parameter	Value
colsample_bytree	0.19088470325102014
subsample	0.9304141034109051
learning_rate	0.477510574908984
num_leaves	115
max_depth	18
min_child_samples	53
reg_alpha	0.2609865674187428
reg_lambda	1.3303484138905937
min_split_gain	0.0007545705453046434
bagging_freq	17
feature_fraction	0.8423132245598192
scale_pos_weight	1.113987259898614
min_child_weight	7.651492399061174
max_bin	786
min_data_in_leaf	52
min_sum_hessian_in_leaf	2.9989911144951256

S16 Table: Hyperparameter values for the false alarm categorical model
