# Supplementary material for: Gradient boosted decision trees reveal nuances of auditory discrimination behavior
Source: PLoS Comput Biol. 2024 Apr 16;20(4):e1011985. doi: 10.1371/journal.pcbi.1011985 (PMC11051626; doi:10.1371/journal.pcbi.1011985)
Supplement: S17 Table — (PDF) [file pcbi.1011985.s024.pdf]

S17 Table

Parameter	Value
colsample_bytree	0.5826037749242697
subsample	0.9632104755468021
learning_rate	0.22727575447213846
num_leaves	45
max_depth	15
min_child_samples	61
reg_alpha	4.265341824464033
reg_lambda	2.3404053166956853
min_split_gain	0.004157693187481298
bagging_freq	6
feature_fraction	0.9019287831358274
scale_pos_weight	1.0694853574196075
min_child_weight	6.892115531076795
max_bin	224
min_data_in_leaf	79
min_sum_hessian_in_leaf	11.443671701974884

S17 Table: Hyperparameters for the miss/hit gradient-boosted decision tree model
