# Supplementary material for: Gradient boosted decision trees reveal nuances of auditory discrimination behavior
Source: PLoS Comput Biol. 2024 Apr 16;20(4):e1011985. doi: 10.1371/journal.pcbi.1011985 (PMC11051626; doi:10.1371/journal.pcbi.1011985)
Supplement: S18 Table — (PDF) [file pcbi.1011985.s025.pdf]

S18 Table

**Parameter**	**Value**
colsample_bytree	0.46168728494506456
alpha	8.758272905706946
n_estimators	82
learning_rate	0.2165288044507529
max_depth	18
bagging_fraction	0.7000000000000001
bagging_freq	0

S18 Table: Hyperparameters for the reaction time gradient-boosted regression tree model predicting the reaction time from the onset of the target word from the subset of correct hit responses.
