# Supplementary material for: Gradient boosted decision trees reveal nuances of auditory discrimination behavior
Source: PLoS Comput Biol. 2024 Apr 16;20(4):e1011985. doi: 10.1371/journal.pcbi.1011985 (PMC11051626; doi:10.1371/journal.pcbi.1011985)
Supplement: S19 Table — (PDF) [file pcbi.1011985.s026.pdf]

S19 Table

Hyperparameter	Value
colsample_bytree	0.9984483617911889
alpha	10.545892165925359
n_estimators	120
learning_rate	0.2585298848712121
max_depth	20
bagging_fraction	1.0
bagging_freq	23
lambda	0.19538105338084405
subsample	0.8958044434304789
min_child_samples	20
min_child_weight	9.474782393947127
gamma	0.1571174215092159
subsample_for_bin	6200

S19 Table: Hyperparameters for the absolute reaction time gradient-boosted regression tree model that predicts the reaction time relative to the female talker type trial start time.
