# Supplementary material for: Gradient boosted decision trees reveal nuances of auditory discrimination behavior
Source: PLoS Comput Biol. 2024 Apr 16;20(4):e1011985. doi: 10.1371/journal.pcbi.1011985 (PMC11051626; doi:10.1371/journal.pcbi.1011985)
Supplement: S20 Table — (PDF) [file pcbi.1011985.s027.pdf]

S20 Table

**Parameter**	**Value**
colsample_bytree	0.5870762820095368
alpha	10.840482953967314
n_estimators	70
learning_rate	0.18038495501541654
max_depth	20
bagging_fraction	0.9
bagging_freq	30

S20 Table: Hyperparameters for the absolute reaction time gradient-boosted regression tree model that predicts the reaction time relative to the male talker type trial start time.
