# Supplementary material for: Gradient boosted decision trees reveal nuances of auditory discrimination behavior
Source: PLoS Comput Biol. 2024 Apr 16;20(4):e1011985. doi: 10.1371/journal.pcbi.1011985 (PMC11051626; doi:10.1371/journal.pcbi.1011985)
Supplement: S21 Table — (PDF) [file pcbi.1011985.s028.pdf]

S21 Table

Ferret ID	Talker Type	Alpha	Bagging Fraction	Bagging Freq	Colsample bytree	Learning Rate	Max Depth	N Estimators
F1702	Female	14.223 322 406	0.9	15	0.437 631 712 494 342 5	0.295 072 885 150 600 2	17	72
F1702	Male	13.683 546 599	0.9	27	0.274 885 701 704 623	0.299 693 972 574 154 14	8	92
F1815	Female	7.971 900 722	0.8	3	0.340 044 537 718 209 1	0.276 768 130 014 741 04	18	94
F1815	Male	8.423 806 867	0.9	17	0.207 299 675 926 581 63	0.156 195 214 112 881	18	70
F1803	Female	12.715 761 382	0.9	3	0.390 041 985 169 617 1	0.299 974 304 007 418 9	17	86
F1803	Male	9.372 039 068	0.9	26	0.336 788 976 012 014 63	0.230 500 554 063 202 1	10	94
F2002	Female	14.643 599 551	0.8	7	0.500 326 655 529 420 5	0.249 352 825 537 214 47	12	96
F2002	Male	7.975 250 677	0.9	1	0.422 911 096 129 035 7	0.261 787 479 450 885 1	20	70
F2105	Female	13.306 184 442	0.9	16	0.244 948 849 315 071 96	0.295 266 603 348 277 37	15	100
F2105	Male	5.599 860 028	0.1	0	0.129 441 717 096 984 3	0.241 117 830 565 032 41	20	42

S21 Table: Hyperparameters for the absolute reaction time models for each ferret ID broken down by Female/Male talker type.
