# Supplementary material for: Gradient boosted decision trees reveal nuances of auditory discrimination behavior
Source: PLoS Comput Biol. 2024 Apr 16;20(4):e1011985. doi: 10.1371/journal.pcbi.1011985 (PMC11051626; doi:10.1371/journal.pcbi.1011985)
Supplement: Attachment — Submitted filename: Response2reviewers.pdf [file pcbi.1011985.s029.pdf]

Dear Dr Theunissen, Dear Reviewers,

We are grateful for the opportunity to revise our manuscript and include below a point by point response to the very constructive feedback we received. In addition to numerous small changes / additions / clarifications we have made 3 substantive ones which are briefly summarised here:

First, we have rewritten the manuscript to make it clear that our primary goal is to introduce this machine learning approach as a suitable tool for the analysis of behaviour, providing additional explanations to (hopefully) grant readers a more intuitive understanding of the methods and the resulting visualisations.

Second, we have included results from mixed effect GLMs to highlight that the key results stand with either method, but that the gradient boosted models offer additional advantages and very useful visualisation tools, particularly for understanding how multiple features interact with each other to ultimately shape behaviour.

Third, we have tried to further unpick the acoustic factors that link words that animals commonly false alarm to. We used various measures of acoustic similarity between target and non-target words and correlated these to the outputs of the gradient boosted regression to ask what measures best correlated with an increased likelihood of false alarms. This analysis suggested that shared spectrotemporal features are most likely to underlie the ferrets false alarms and demonstrated that neither word duration or temporal envelope were predictors of false alarm behaviour.

Below, we address specific points from the reviewers. We provide line numbers which refer to our uploaded document in which figures are embedded (available ± track changes, although the changes we have made are so numerous that we suspect this will not be a terribly useful document for reviewers!).

Comments to the Authors:
Reviewer #1: In this study, Griffiths et al. Use a machine learning algorithm, gradient boosted trees, to model behavioral data from ferrets performing a word detection task at a trial-wise level. They both trial outcomes and response times of ferrets' responses to natural and F0 shifted speech with reasonable success. Importantly, they use ML tools to interpret these models to determine the relative importance of various parameters from behavioral performance. This leads to both confirmatory and novel insights.

Overall, this is a well-executed study that tackles a subject that has received scant attention in the auditory field – detailed modeling of behavioral responses. More generally, these modeling techniques can be used across modalities and species, and should be of wide interest to behavioral neuroscientists. While I overall support the eventual publication of this study, I have a few suggestions for improvement that I hope the authors will find constructive.

We thank the reviewer for their interest in our work and their constructive suggestions.

Major comments:

1) The authors consider a number of factors affecting performance, but I was surprised to not see temporal factors considered, e.g. the duration of the word, number of syllables, or envelope similarity. These may help explain why the animals are able to maintain performance across varying F0s (if target duration is significantly different from non-target durations) or conversely, why they false-alarm more to specific words (similar durations/envelopes?). Considering the primacy of temporal cues in speech perception, this would be worth modeling!

We are grateful for the reviewer pushing us to do this analysis as we feel it has provided valuable insights into the animals' behaviour. To explore the features that might underlie the animals' false alarm pattern we considered three types of measures; first, we used a cochleagram model to estimate the representation of each token at the auditory periphery (with the caveat that this is a human model, and therefore likely overestimates the frequency resolution available to the ferrets). Second, we extracted the envelope of the amplitude waveform in order to explore the role of the temporal envelope. Third, we considered the difference in duration of each word token and the target word. For the first and second measure we compared the target and each non-target word token (for all tokens from the same talker) using by computing a Pearson's correlation, aligning the tokens at their onset. We also used a cross-correlation approach acknowledging that we don't *a priori* know which elements of a given token animals might confuse (e.g. we might imagine the "idence" of "confidence" might be more readily confused with "instr" than with "uments"). We took the maximal cross correlation for all possible target-distractor pairs, although similar results were achieved taking the average. From these correlation / cross correlation scores we could then rank words according to their similarity with the target:

For the cochleagrams (left correlation, right cross correlation)

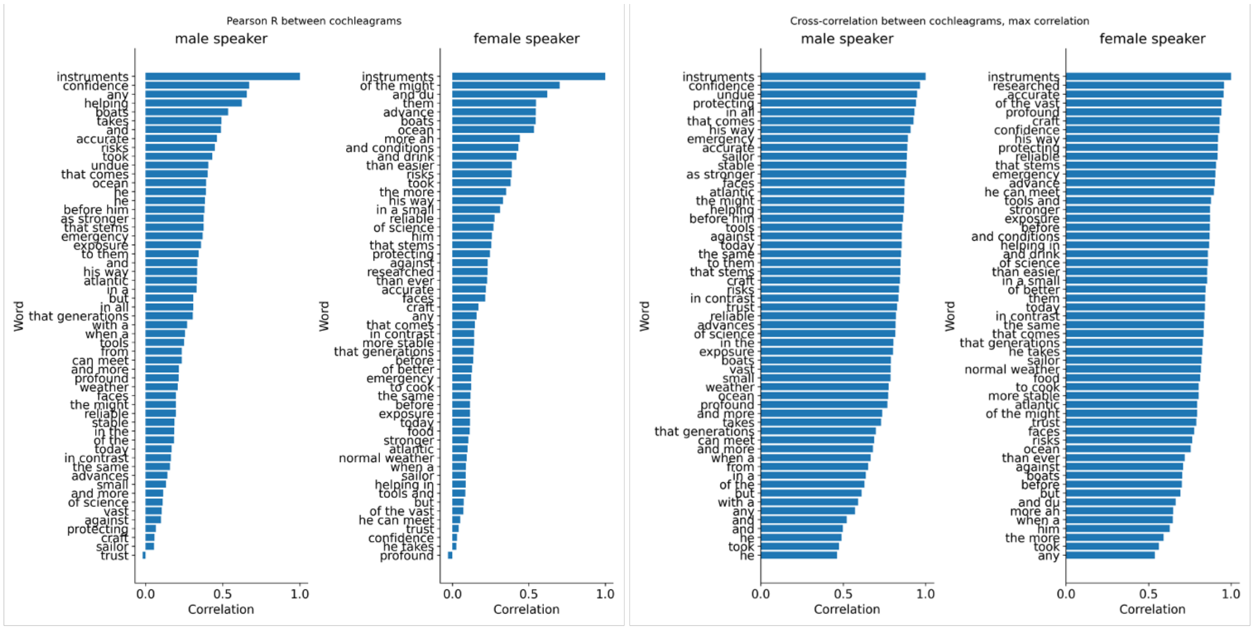

For the wave forms:

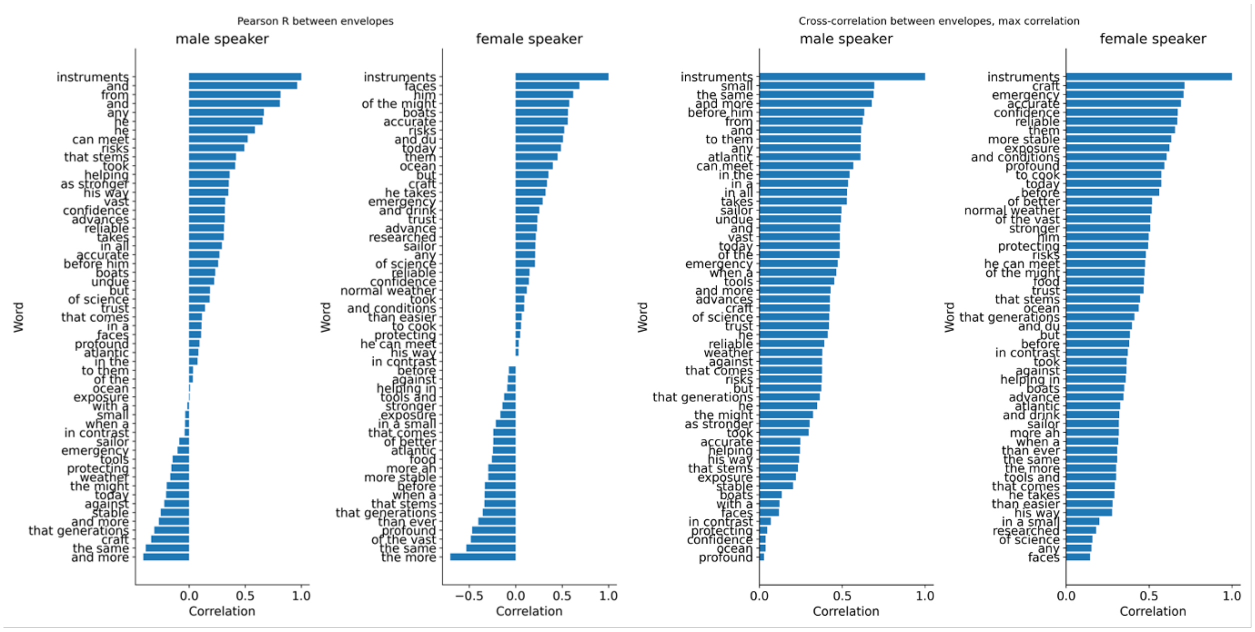

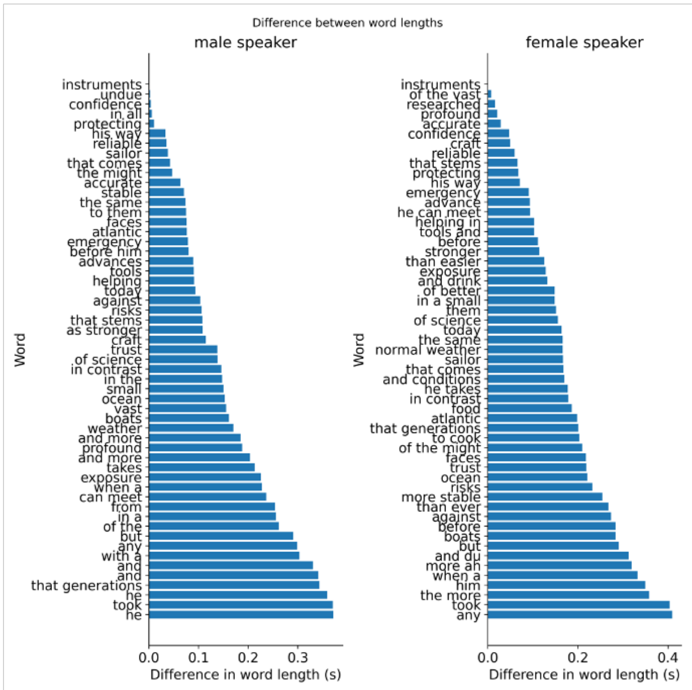

Finally, we took the duration of each word token, assuming that the animals used the silent gaps between words to mark token onset (anecdotally when we briefly introduced a bug in the stimulus presentation code that eliminated this gap ferrets' performance plummeted, so this seems a reasonable assumption).

To compare the predictive power of each, we combined the data from male and female talkers, and calculated the Spearman's correlation coefficient between the permutation importance and each statistic. The maximum cross-correlation between the cochleagram provided the strongest relationship (spearman's r = 0.529), explaining 28% of the variance in the animals' behaviour. Difference in word duration also had a significant relationship with permutation importance (r = 0.424), but this relationship is in the opposite direction of that that would be predicted (greater duration differences predict a greater likelihood of false alarms). Words with highest permutation importance can be seen to span a range of duration differences further confirming the observation that in all likelihood similar duration is not a cue that the ferrets are relying upon to solve the task. Neither of the amplitude waveform measures produced statistically significant relationships. From this we therefore conclude that animals are relying most heavily on spectrotemporal features of the word to perform the task.

We have included a new figure in the main manuscript (Figure 6) that reports these findings:

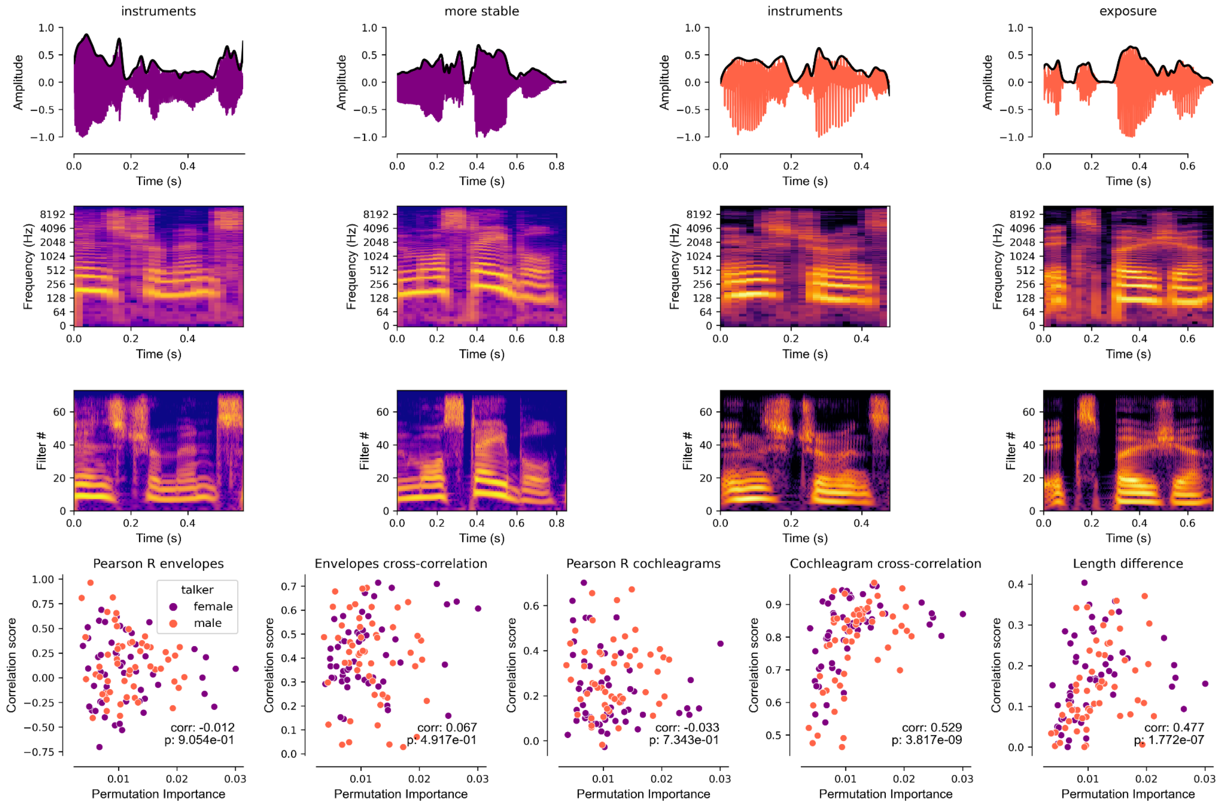

Figure 6 Spectrotemporal similarity predicts false alarm likelihood

A, top to bottom: waveform, spectrogram, and cochleagram of instruments for the female talker stimulus. The black line in the waveform plot indicates the extracted envelope. B, top to bottom: waveform, spectrogram, and cochleagram of `more stable', one of the words that were associated with a high chance of response in our absolute reaction time model for the female

talker. C, same as A but for the male talker stimulus. D, same as B but for the word `exposure', which was associated with a high rate of response in our male talker absolute reaction time model. E, the pearson's r correlation between the envelopes of the non-target words relative to the target over each non-target word's respective permutation importance. F, the maximum cross-correlation coefficient between each non-target word and the target word over each non-target word's respective permutation importance. G, same as E but using the cochleagram representations of the target and non-target words rather than the envelopes. H, same as F but for the cochleagram of each non-target word relative to the target word rather than the envelope. I, the absolute difference in duration (length) between each non-target word and the target word over its respective permutation importance.

2) In Fig. 1D, are the labels for inter- and intra-trial F0 changes correct? It appears that inter-trial F0 changes induce both lower p(correct) and higher p(FA) leading to decreased performance (which may not be significant), suggesting that pitch streaming is not providing a behavioral advantage. This seems somewhat contradictory to the claim that there is an advantage to pitch streaming in the abstract and elsewhere. Faster RTs may be the case (Page 6) but this is not necessarily advantageous if performance is decreasing.

The labels here are correct; we now provide (basic) statistics on these measures [Lines 94-108] repeated measures ANOVAs with factors talker and pitch roving type [control / inter / intra], with follow up pairwise comparisons where appropriate (see below, supplementary tables S1-S6) show that for hits, while there is a significant talker * rove interaction, there are no significant pairwise comparisons.

Within-group factor	SS	Degrees of freedom (numerator)	Degrees of freedom (denominator)	MS	F-value	Uncorrected p-value	GG corrected p-value	Generalized eta-squared	GG epsilon factor
roving_type	0.0002851	2	8	0.00014255	0.1673	0.8488	0.8339	0.0037	0.9247
talker	0.050970082	1	4	0.050970082	12.0173	0.0257	0.0257	0.3979	1
roving_type * talker	0.0215	2	8	0.0108	16.3772	0.0015	0.0099	0.2181	0.5936

Supplementary Table S1. Repeated-measures ANOVA for the hit statistic with roving type and talker as factors.

A	B	mean(A)	mean(B)	diff	se	T	p-tukey	hedges	talker
control	inter	0.8280	0.8823	-0.0542	0.0390	-1.3916	0.3756	-0.7149	Female
control	intra	0.8280	0.8738	-0.0458	0.0390	-1.1752	0.4893	-0.6126	Female
inter	intra	0.8823	0.8738	0.0084	0.0390	0.2164	0.9746	0.1648	Female
control	inter	0.8201	0.7508	0.0693	0.0324	2.1367	0.1239	1.0451	Male
control	intra	0.8201	0.7659	0.0542	0.0324	1.6725	0.2551	1.3949	Male
inter	intra	0.7508	0.7659	-0.0151	0.0324	-0.4642	0.8891	-0.2455	Male

Supplementary Table S2. Pairwise Tukey HSD posthoc test statistics for the hit statistic comparing the roving types for each talker type.

For false alarms there is pairwise posthoc tests revealed that for trials using the female voice false alarms were significantly higher in both roved conditions that control conditions, but were not significantly different from each other.

Within-group factor	SS	Degrees of freedom (numerator)	Degrees of freedom (denominator)	MS	F-value	Uncorrected p-value	GG corrected p-value	Generalized eta-squared	GG epsilon factor
roving_type	0.0347	2	8	0.0174	32.541	0.0001436	0.0011539	0.3932	0.6985
talker	0.0057	1	4	0.0057	7.7165	0.04993	0.04993	0.0961	1
roving_type * talker	0.0087	2	8	0.0043	16.3283	0.0014991	0.0082412	0.1391	0.6338

A	B	mean(A)	mean(B)	diff	se	T	p-tukey	hedges	talker
control	inter	0.1213	0.2395	-0.1181	0.0288	-4.0986	0.0039	-2.2667	Female
control	intra	0.1213	0.2069	-0.0855	0.0288	-2.9677	0.0294	-2.2529	Female
inter	intra	0.2395	0.2069	0.0326	0.0288	1.1310	0.5143	0.5526	Female
control	inter	0.1969	0.2445	-0.0476	0.0309	-1.5418	0.3072	-0.8621	Male
control	intra	0.1969	0.2089	-0.0120	0.0309	-0.3884	0.9207	-0.2324	Male
inter	intra	0.2445	0.2089	0.0356	0.0309	1.1533	0.5016	0.6446	Male

Supplementary Table S4. Pairwise Tukey HSD posthoc test statistics for the false alarm statistic comparing the roving types for each talker type.

Within-group factor	SS	Degrees of freedom (numerator)	Degrees of freedom (denominator)	MS	F-value	Uncorrected p-value	GG corrected p-value	Generalized eta-squared	GG epsilon factor
roving_type	0.4941	2	8	0.2470	19.0512	0.0009067	0.0016395	0.2171	0.8836
talker	1.5566	1	4	1.5566	13.3321	0.0217	0.0217	0.4662	1
roving_type * talker	0.0379	2	8	0.0190	1.4193	0.2968	0.3006	0.0208	0.5931

Supplementary Table S5. Repeated-measures ANOVA for the d' statistic with roving type and talker as factors

A	B	mean(A)	mean(B)	diff	se	T	p-tukey	hedges
control	inter	1.9759	1.6675	0.3084	0.1582	1.9500	0.1443	0.8036
control	intra	1.9759	1.7690	0.2070	0.1582	1.3086	0.4028	0.5891
inter	intra	1.6675	1.7690	-0.1014	0.1582	-0.6414	0.7987	-0.2727

Supplementary Table S6. Pairwise Tukey HSD posthoc test statistics for the d' statistic comparing the roving type.

For overall d' there is a significant effect of talker and roving type, with no interaction, but again the pairwise posthoc comparisons do not show any significant differences. We therefore interpret this as consistent with our results from the gradient boosted decision trees (and also our GLMs) which show no effects on performance but a significant acceleration of reaction times when the word preceding the target was the same pitch value.

The text has been revised as follows:

To understand whether ferrets form a pitch-tolerant representation of the target word, we considered the impact of F0 changes on performance (Fig 1D-F). Two-way repeated measures ANOVAs with factors talker (male/female) and rove type (control / inter / intra) showed that for hit rates there was a significant effect of talker and significant talker x rove interaction, but no significant pairwise comparisons across pitch roved conditions (supplementary tables 1 and 2). For false alarms there was again a significant effect of talker, rove and talker x rove interaction, with posthoc comparison showed that for the female talker control F0s elicited significantly lower false alarm levels than either rove type, but that the rove types were not significantly different from each other (supplementary tables 3 and 4). For sensitivity (d') measures there were again significant effects of talker and rove type, but post hoc comparisons showed no rove conditions to be significantly different from each other (supplementary tables 5 and 6). Therefore overall, while subjects were better on female talker trials than male talker trials, the performance on inter and intra-trial roved trials was largely equivalent (Fig.1D-F). When the performance was broken down according to the actual F0 value we observed there was a modest influence of F0 on hit rates, such that the highest hit rates were observed for the female talker's up-shifted F0 trials (Fig.1H). False alarms, in contrast, were lower for the trained F0 values for both the male and female talkers.

3) Writing in abstract and introduction: The title of the paper suggests that the paper is going to be about using ML to model behavioral data. But the beginnings of 1) the abstract emphasizes pitch perception, 2) the summary on hearing loss and brain mechanisms, and 3) the introduction on auditory cortex and inactivation studies. Overall, this results in a confusing entry into the story. I would suggest that the authors reframe these sections by focusing on the need to model behavioral data, other models that are out there (e.g., drift diffusion models or LATER models), what ML approaches can offer etc. The details can of course be there, but not having them in stress positions would help alleviate this confusion.

On reflection we agree with the reviewers on this point and have considerably reworked the introduction, discussion and significance statement to better reflect the novelty and utility of the modeling aspects, in particular the advantages offered be the machine learning approaches and their accompanying visualization methods.

4) The paper is highly technical and not easily accessible to biologists. Some explanation is provided in the methods, but I would have liked a more didactic paragraph in the introduction on gradient boosted trees and SHAP plots, and some help interpreting these plots in the Results section.

We apologise for that, as discussed above, as suggested we have now included a section in the results devoted to an explanation of gradient boosted models and the visualisation methods that enable their interpretation. Additionally we have reworked the manuscript throughout to try and make it an easier read for non-experts:

Introduction to gradient boosted decision trees [Lines 117-171]

Gradient boosting is a supervised machine learning algorithm used for classification and regression problems and is particularly advantages due to the tools available to visualise how a model exploits information to perform the task. The basic principle is that decision trees are built by splitting observations based on feature values, with the algorithm seeking and selecting split that results in the highest gain in information by comparing predicted outcomes to observed ones.   We chose this machine learning approach as our data is abundant in sample size and tabular. While its application to animal behavioral work is to our knowledge novel, this scenario of structured, dense data is ideal for gradient-boosted decision trees, as this type of method has often been used in recommender systems (Luo et al., 2022) as well as economic predictive modeling for human behavior in customer loyalty (Machadoet al., 2019). A machine learning approach is ideal because it can uncover non-linear dependencies in the data and without users being required to predetermine interaction effects in their model. Moreover we can consider multiple stimulus features, such as the talker and pitch of the word, as well as the trial history parameters (was the previous trial correct, was the previous trial a catch trial) and non-stimulus features (such as the timing of the trial within the session, the time of the target word within the trial, and the side that the animal was required to respond) that may influence performance but do not necessarily inform our experimental hypothesis.

We used lightGBM (Ke et al., 2017) to implement a gradient-boosted machine (GBM) approach. We considered two types of models – decision tree models that performed categorical discriminations, for considering whether responses to targets were misses or hits, and whether responses to catch trials were false alarms or correct rejections, and decision-tree regression models to predict continuous reaction time data. In each case, we trained models using 5-fold cross validation and used held out data to report both the accuracy and balanced accuracy (which is particularly helpful for data in which observations are unequal in number between categories and where accuracy may therefore be overinflated). To assess which variables were utilised by the model we used two metrics; feature importance and permutation importance. The GBM decision and regression tree method consists of many decision trees and features will potentially be used many times to split the data; to understand the contribution of a feature the gain provided must be aggregated across trees. Therefore the feature importance metric assesses how a given feature improves the models accuracy by summing the gain provided by that feature across all of the times that its used in the model. A higher gain implies that the feature is more important for generating predictions. In lightGBM the loss functions (from which gain is computed) are the mean squared error (MSE) for regression tasks and the log loss for classification task. Its units are the same as the target variable, seconds, and its upper and lower bounds are minus to positive infinity. Permutation importance provides a complementary measure of the importance that any given feature provides to the model. The permutation

feature importance is the decrease in a model score when a single feature is randomly permuted. The higher the permutation importance, the larger the contribution a variable makes to the model; a score of 0.1 for a model with 70\% accuracy reflects a drop to 60\% accuracy for a classification problem. One caveat with the permutation importance is that it assumes that all variables are independent so can underestimate the contribution of a given variable in some circumstances (Molnar et al., 2023).

To visualise the way in which variables impacted model predictions, and how variables interact with one another we used SHapely Additive exPlanations (SHAPs) which are a common way of understanding machine learning models based on Shapely values. Shapely values were derived from cooperative game theory and represent the average contribution of each feature to all possible combinations of features (Lundberg et al., 2017). SHAPs extends this to machine learning models; for every feature and every observation in the training set we obtain a SHAP value and therefore there are as many SHAP values as there are observations. For a classification task the SHAP values are expressed as the log(odds) so can be directly interpreted as the impact of a given feature on the probability e.g. of making a miss. For our regression models the SHAP scores are the impact on reaction times, expressed in seconds. Here we use SHAP summary plots to provide intuitive and interpretable visualisations of the effects of all variables in a model, and partial dependency plots to visualise combinations of features of interest. The partial dependency plots are particularly helpful for understanding how, for example, behaviour varies across individual subjects, and for examining the potentially non-linear interactions between features that the model has learned to exploit.

Minor:
Fig.1: Could you use different colours to signify the trial types in Figs. 1D-G and F0 value in Fig. 1H?

We have done this as requested.

Figs 2 -5: Could you please standardze the font size used for labelling axes? Some are too small to be readable.

Apologies for this, we have modified this throughout.

Page 10 Line 318 Please provide a reference or description of the STRAIGHT pitch shifter

Thank you for flagging this omission; we have now added a reference to Kawahara 2006, and some additional explanation [line].

Additional citations featuring behavioral responses in animals to F0 shifts: Osmanski and Wang, PNAS 2023, Kar et al., eLife, 2022 (also includes a model), Bregman et al., PNAS 2016, Neilans et al. Plos ONE 2014.

We have included these in relevant sections of the discussion [lines].

Reviewer #2:

This manuscript develops a novel application of a machine learning tool to the analysis of complex auditory behavior. The authors use gradient boosted decision trees and -regression to analyze data from ferrets during a word detection task, in which the timing, location, speaker and pitch of target and distractor words varies across trials and sessions. The analyses provide a quantitative measure of the relative importance of different variables to hit/miss, false alarm, and reaction time outcomes, confirming the impact of some variables identified using more traditional analysis. The analysis supports the authors' hypothesis that ferrets are mostly able to generalize categorical representation of words across different voice pitch. There are some other interesting observations, e.g., speaker identity influences hit rate but not false alarm rate.

This work takes a novel approach to analyzing an interesting new behavior. The authors make a reasonable case that gradient boosted tree methods address limitations of more traditional methods for analyzing behavior, and they demonstrate that these methods can be applied to their behavioral data. However, the novelty of the method and the specific questions about behavior seem muddled, raising questions about the value of this fairly complex analysis. These are probably addressable--largely by addressing question around interpretation of effect size in the new models' results and a meaningful comparison with simpler, traditional methods.

MAJOR CONCERNS

1. What is this manuscript about? Reading the title, one might imagine that it is intended to validate a new method for analyzing behavior. But reading the introduction and discussion, it sounds like gradient boosted decision trees are established, and the questions are focused on specific variables related to a specific aspect of auditory processing. The results are somewhere in the middle, emphasizing the novelty of gradient boosted trees, but not really demonstrating when they are able to show things that more traditional methods of behavioral analysis don't show. In the Methods, the authors cite "Linear mixed effect and generalized linear models" (L. 353) as established approaches. The baseline analysis in Fig. 1 seems much simpler than these norms, only considering one or two variables at a time and not really pushing the traditional methods. A simple linear classifier could be trained to predict outcomes like hit vs. miss, and it seems like the weight of each variable in the classifier could be compared directly to the decision tree statistics. Without this comparison, a reader finds themselves wondering why they use the more complex and opaque method proposed here. It would help if either the authors could provide a quantitative contrast with a traditional method OR rewrite some key parts of the manuscript to make it clear that the approach does not need validation.

As noted above in response to both the Editor, we have considerably rewritten the manuscript to address the first half of this comment, and have included a full analysis using mixed effect models to address the second half of this comment.

Specifically, to provide a robust and fair comparison to 'traditional' mixed effects models we ran mixed effects generalised linear models to predict miss/hits and false alarms/correct rejections (both binomial models with a logit link) and linear model to model reaction times. To provide a fair comparison we used the same 5 fold cross validation to assess model performance on held-out data, and report the average coefficients across the cross validated models. Overall the GLMs support the conclusions of the gradient boosted decision tree approach, but provide similar or substantially worse predictive power and much more limited scope for understanding interactions.

We have added the following text and one main and one supplemental figure [lines 356-]:

Comparison to traditional linear mixed-effects models

To compare the average performance of our gradient-boosted tree models with traditional linear-mixed effects models, we ran linear mixed-effects models on the variables used in our corresponding gradient-boosted regression tree models with ferret as a random (group) effect using the statsmodels package in Python. Like our gradient-boosted trees, we implemented five-fold cross-validation for a fair comparison. In most cases, our gradient-boosted decision and regression tree models were better or comparable to the linear mixed effects models. Reassuringly, many of the same statistical main effects were found with both approaches. However, there were some specific instances in which the GBM approach was superior (described below), and the available tools for the visualization of partial dependencies offered the advantage that the non-linear interactions between features could be meaningfully explored and quantified using SHAP values.

A mixed effects GLM (binomial, logit link) predicting hit vs. misses had an accuracy of 62.47% for the train split, 62.12% for the test split (this was comparable to the gradient-boosted regression trees, where the model had an average train balanced accuracy of 62.17% and an average test balanced accuracy of 62.09%). Mirroring the GBM, significant coefficients were returned for a talker, audio side, and target pitch (for 191 Hz vs reference of 109 Hz, Fig.7A). Neither the ferret ID, trial number nor target time parameters returned by the GBM were returned as significant by the mixed effects model (Tables S7,S8).

A mixed effects model predicting false alarms during catch trials had lower accuracy than the corresponding GMB model (balanced accuracy was 54.90% on the train data set vs. 61.5% and 54.70\% on the test dataset compared to 61.46%). The mixed effects GLM returned significant coefficients for F0 (all values vs reference 109Hz), the previous response being correct, and the trial number (Fig.7B, Tables S9,S10). The GBM additionally assigned feature importance to the time within the trial, ferret ID, and whether the trial was intra-trial roving (Fig.3).

A mixed effects model predicting the reaction time for correct hit responses from behavioural variables had a mse of 0.091s for the train dataset, 0.092s for the test dataset, which was comparable to the mse of the gradient-boosted regression tree model (train mse = 0.092s, train mse =0.102s). Given the restriction that reaction times are between 0-2 s (meaning there are few outliers and a relatively normal distribution), this is perhaps not surprising. The model

recapitulated the effects of the GBM, returning significant coefficients for talker (faster to female voice), F0 (124 Hz faster than 109 Hz), trials in which the precursor and target had the same F0 were faster than those in which they differed, reaction times were faster for targets later in the trial and for later trials in the session (Fig.7C, Supplemental Tables S11,S12). While the key results were the same across analysis approaches, the ability to visualize SHAP scores for all observations from each animal across multiple variables still provides additional clarity, which could be advantageous when trying to relate brain and behavior. For example, Figure 4D shows how target F0 impacts reaction time for each individual ferret, showing opposite patterns in F1702 and F2105, something that would not be apparent with the mixed effects model coefficients.

Where the linear model failed was in predicting the absolute release time solely based on which words were in a trial. To match the GBM approach, we used ordinary least squares, which, like the GBM, did not consider ferret as a factor, and again separated male talker and female talker trials to generate two models. The mse for the OLS model was nearly an order of magnitude larger than the GBM model, 0.15 and 0.19s, respectively for the male and female talker models, compared to errors of 0.0193s and 0.049s for the female and male talker for the GBM (Fig.S5C, Supplemental Tables S12,S13). Critically, the size of the coefficient for 'instruments' was barely greater than for the first-ranked non-target word in either model. Although there was some similarity in the ranking of non-target words between the linear regression and the GBM, the low overall model accuracy would make it hard to confidently make conclusions about false alarm behavior based on the linear regression alone. This analysis highlights that the GMB model has an advantage when predicting outlier behavior; false alarms to individual non-target word tokens are inherently rare in trained animals, and there is not a fixed response latency (as shown in the reaction time analysis) even if we can assume that animals trigger responses to the onset of word tokens (which the strong relationship between false alarms and cochleagram cross-correlation but not between correlation coefficients suggests is not the case). When performing the response time analysis with the GBM, we subsampled data to ensure that word frequency could not erroneously bias the resulting models; however we repeated the modeling with the original (non-uniform) distribution of word frequencies and the resulting permutation importance scores for non-target words were highly correlated Fig.5C, Spearman's R = 0.72 and 0.87 for female and male talker models respectively) suggesting that this subsampling was unnecessary.

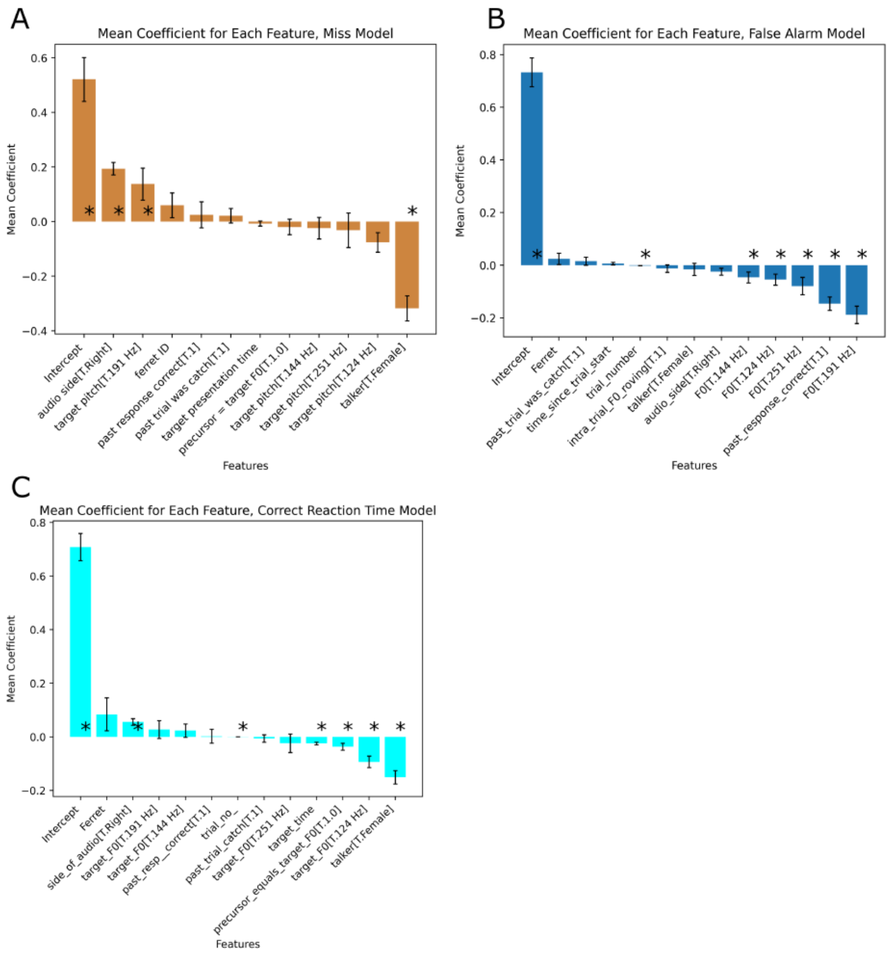

Figure 7. Mixed effects models show equivalent or worse performance Average coefficients values for the mixed effects model predicting A, a miss response for a target trial, B, a false alarm for a catch trial, and C, the reaction time during a correct target trial. Reference talker: male talker, reference F0: 109 Hz, reference side of audio: left side. Asterisks represent mean p-values < 0.05. Error bars represent the mean standard deviation.

2. "feature importance, … SHAP feature values" (L 115.). "SHAP feature importances" (Fig. 2B) "SHAP partial dependency" (Fig. 2C). It's clear that the relative size of these statistics provides a measure of how much the corresponding variables are able to explain behavioral outcomes. However, the units and magnitude of the various effects are not obvious. Digging into the methods, the reader learns that these are "Shapley Additive Importance features" (L. 408) but not much else. To be more specific: What does a step from 4500 to 5000 in Fig. 2A mean? Why is the scale to 55000 in Fig. 3A? Can an x value in Fig. 2B be interpreted as a probability? What are the shaded boxes in Fig. 2C? Or the meaning of the individual dots? (single trials?) The words in the text provide a high-level interpretation, but a better explication of the numbers is needed. A place to start would be to define statistics in the Results and provide meaningful units for the plots. Ideally, these would translate to human-interpretable units that give a sense of the

magnitude of impact on behavior outcomes. In more traditional linear analysis, a useful concept, for example, is variance explained. That may or may not be possible with the method used here, but some explication seems necessary.

We hope that our new section introducing gradient boosted models addresses these points:

Introduction to gradient boosted decision trees [Lines 117-171]

Gradient boosting is a supervised machine learning algorithm used for classification and regression problems and is particularly advantages due to the tools available to visualise how a model exploits information to perform the task. The basic principle is that decision trees are built by splitting observations based on feature values, with the algorithm seeking and selecting split that results in the highest gain in information by comparing predicted outcomes to observed ones.   We chose this machine learning approach as our data is abundant in sample size and tabular. While its application to animal behavioral work is to our knowledge novel, this scenario of structured, dense data is ideal for gradient-boosted decision trees, as this type of method has often been used in recommender systems (Luo et al., 2022) as well as economic predictive modeling for human behavior in customer loyalty (Machadoet al., 2019). A machine learning approach is ideal because it can uncover non-linear dependencies in the data and without users being required to predetermine interaction effects in their model. Moreover we can consider multiple stimulus features,such as the talker and pitch of the word, as well as the trial history parameters (was the previous trial correct, was the previous trial a catch trial) and non-stimulus features (such as the timing of the trial within the session, the time of the target word within the trial, and the side that the animal was required to respond) that may influence performance but do not necessarily inform our experimental hypothesis.

We used lightGBM (Ke et al., 2017) to implement a gradient-boosted machine (GBM) approach. We considered two types of models – decision tree models that performed categorical discriminations, for considering whether responses to targets were misses or hits, and whether responses to catch trials were false alarms or correct rejections, and decision-tree regression models to predict continuous reaction time data. In each case, we trained models using 5-fold cross validation and used held out data to report both the accuracy and balanced accuracy (which is particularly helpful for data in which observations are unequal in number between categories and where accuracy may therefore be overinflated). To assess which variables were utilised by the model we used two metrics; feature importance and permutation importance. The GBM decision and regression tree method consists of many decision trees and features will potentially be used many times to split the data; to understand the contribution of a feature the gain provided must be aggregated across trees. Therefore the feature importance metric assesses how a given feature improves the models accuracy by summing the gain provided by that feature across all of the times that its used in the model. A higher gain implies that the feature is more important for generating predictions. In lightGBM the loss functions (from which gain is computed) are the mean squared error (MSE) for regression tasks and the log loss for classification task. Its units are the same as the target variable, seconds, and its upper and lower bounds are minus to positive infinity. Permutation importance provides a complementary measure of the importance that any given feature provides to the model. The permutation

feature importance is the decrease in a model score when a single feature is randomly permuted. The higher the permutation importance, the larger the contribution a variable makes to the model; a score of 0.1 for a model with 70\% accuracy reflects a drop to 60\% accuracy for a classification problem. One caveat with the permutation importance is that it assumes that all variables are independent so can underestimate the contribution of a given variable in some circumstances (Molnar et al., 2023).

To visualise the way in which variables impacted model predictions, and how variables interact with one another we used SHapely Additive exPlanations (SHAPs) which are a common way of understanding machine learning models based on Shapely values. Shapely values were derived from cooperative game theory and represent the average contribution of each feature to all possible combinations of features (Lundberg et al., 2017). SHAPs extends this to machine learning models; for every feature and every observation in the training set we obtain a SHAP value and therefore there are as many SHAP values as there are observations. For a classification task the SHAP values are expressed as the log(odds) so can be directly interpreted as the impact of a given feature on the probability e.g. of making a miss. For our regression models the SHAP scores are the impact on reaction times, expressed in seconds. Here we use SHAP summary plots to provide intuitive and interpretable visualisations of the effects of all variables in a model, and partial dependency plots to visualise combinations of features of interest. The partial dependency plots are particularly helpful for understanding how, for example, behaviour varies across individual subjects, and for examining the potentially non-linear interactions between features that the model has learned to exploit.

We apologise for not labeling plots more clearly in the first instance; all SHAPs value plots are now labeled as log(odds) which is an interpretable measure that can be compared across models to determine the impact of a given variable on the model output. The scores for feature importance are unbounded as it will depend on the decision tree that is created which will have different numbers of splits for each model. The model accuracy calculated on held out data is equivalent to variance explained and we have calculated this for both the GBMs and the linear models.

2a. Currently each analysis gives a list of results, but it is hard to get a sense of the “nuances” revealed by the three different models. Something that might be helpful would be a comparison of relative effect size/importance for each variable between the models. Eg, could a scatter plot contrast the effect of all the model variables (talker identity, animal, side, pitch shift type, etc.) on hit/miss vs. their effect on FA rate? If not a new figure, some of side-by-side discussion would be helpful. The authors do point out that the same variables cannot be included in each model, so it makes sense that a complete direct comparison may not be possible.

We hope that the fact that everything is now expressed as the log(odds) facilitates direct comparison. We considered plotting a giant feature importance plot across models but in the end what we feel is the key ‘selling point’ of these models is actually the ability to look at interactions through the partial dependency plots rather than the main summary plots (which can already be compared side by side) – and without recapitulating all the plots we already had we couldn’t see how to do this.

LESSER CONCERNS

(L. 46) "20,487 trials " It would be helpful to indicate the number of animals here too.

We have added this.

(L. 56) What was the range of trial lengths, i.e., distribution of time from trial start to target onset?

These are detailed in the methods (line 490) - the minimum target time was 0.5 s and the maximum time at which the target could occur was 6.5 s, with target times drawn with a uniform distribution across this range. Catch trials matched these statistics.

(L. 60) "Single male and single female" Does "single" mean one value of F0 per voice?

The training speech set was drawn from recordings made from two talkers (one male, one female). Therefore these two talkers each had a single average F0, although of course there is natural variation around that, which was maintained when pitch shifting (i.e. the talkers were not monotonized).

(L. 61) "trials were introduced in which the F0…" Can the authors provide a sense of how large a portion of trials were used to probe pitch generalization? It's not clear if these trials were probes in the traditional sense or if the animals could learn that multiple different pitches could be target sounds. Did performance on the variable pitch trials change over time?

As noted below in response to R3, given the delays that covid had already imposed we were unable to introduce these trials as probe trials to understand the animals innate responses. Instead trials were equiprobably presented on the inter-trial roving (i.e. 1/3rd of trials were trained talkers, 2/3rds were pitch shifted) and between 50% and 100% of all trials were roved on the intra trial roving. Performance was stable over time once performance on control trials had normalised (when the rules of the task are initially changed in almost all of the tasks we train in the lab animals tend to have a brief period in which they go on strike, performing many fewer trials and performing the trials that they were previously competent in very poorly. The data we include here is from the point at which those two metrics recover, and from that point performance is stable (but the animals have been exposed to at least a few 100 trials).

(L. 79) "trained F0 values" What are the trained F0 values? Not clear in the figure (1H?)

We have changed this to 'control' F0 values in the text to match the figure.

(L. 108, L. 136) The choice of what independent variables to include in which analysis is not always clearly motivated. Is "precursor=target F0" (Fig 2) different from "intra-trial F0 roving" (Fig. 3)? Or are they the same thing? It would help to identify what variables are shared across models and what variables are (out of necessity) different.

We have clarified this - they are nearly the same thing, but on some intratrial roving trials, by chance, the target and preceding non-target word could share the same F0, this variable excludes those trials from the intra-trial roved F0.

[line 174-180]

The variables provided to the model were: the talker (male/female), the side (left/right) of the audio presentation, the trial number (in the session), the subject identity (ID), target presentation time (within the trial), the target F0, whether the previous trial was a catch trial, whether the previous response was correct, and whether the F0 of the non-target word preceding the target matched that of the target (non-target F0=target F0, this selects intra-trial roved trials eliminating those trials where by chance the word before the target matched the target F0).

(L. 115) "SHAP" please define here.

This is defined in the new section on Gradient boosted models

(L. 165) "mean squared error ... was 0.0947" What is the baseline error, e.g., for a completely random model? Can this be translated into percent correct, as for the hit/miss model?

Thank you for suggesting this. To address the question of what the baseline error is we randomly shuffled trial variables and reaction times 1000 times and calculated the mean squared error, using the original model's hyperparameters. This allowed us to estimate baseline performance for both the reaction time model and the response time model that was used to detect false alarms:

For reaction times [line 240]: *Our test-set mean squared error (mse) using 5-fold cross-validation was 0.102s compared to a noise floor (calculated by randomizing the relationship between trials and reaction times) test mse of 0.133s (train mean-squared error = 0.092s, compared to a noise floor train mse of 0.105s).*

For response times [line 279]: *The prediction accuracy of this model was excellent for both talker types, with a test mse of 0.0193s for the female talker compared to a noise-floor test mse of 1.804s (see methods) and a train mse of 0.0189s compared to a noise-floor train mse of 1.792s. The test mse for the male talker was 0.0499s compared to a noise-floor test mse of 1.959s, with a train mse of 0.0493s compared to a noise-floor mse of 1.949s (5-fold cross-validation for both train and test metrics).*

(L. 168) "whether the F0 of the previous word equaled the target word" Is this the same as inter- vs. intra-trial roving?

This has been clarified as discussed above.

(L. 196). "test mse…" Again (as L. 165), can a noise floor be provided? Understandably, percent correct doesn't make sense here.

We have addressed this above.

(L. 204) "The commonly false alarmed words…" A cursory inspection of spectrograms of the high-FA words suggests that they do share structure with the target word. Can the authors perform a comparison between spectrograms? For example, one could compute the correlation, point-by-point between the target and confused word spectrogram for the same voice.

We have addressed this fully in response to R1 and believe these extra analysis are a valuable addition to the manuscript.

(L. 269) What was the overall sound level of the stimuli?

Sounds were calibrated to be 65 dB SPL, as noted in the methods [line 478].

(L. 306) "3/5 animals…" did training history predict animal-specific effects in the model analysis?

Not really, for example figure S2C three animals (including both F2002 and F2105 who were trained from day 1 with two talkers) had slightly higher miss rates than two others. Other partial dependency plots reveal F2105's behaviour to be idiosyncratic (e.g. she was the only animal to have a lower FA rate for the male talker, and had a lower overall FA rate). With such a limited sample size its very difficult to draw any reasonable conclusions.

(L. 383) The authors appear to have carefully controlled sampling to avoid potential bias. They then report that results were the same as if they didn't subsample. Given the non-parametric nature and flexibility of the gradient boosted tree method, is balancing independent variables necessary? One might think that the method is intrinsically able to account for these factors. A reference indicating why or why not these preprocessing steps were necessary would be helpful.

We performed the subsampling as this is the gold standard approach for 'traditional' models. However the reviewer is right and one of the advantages of these methods is that they are much more robust to such factors; a point we now make more strongly in the manuscript by showing the permutation importance scores for the model with and without subsampling, which are highly correlated: (supplementary figure 5C).

C

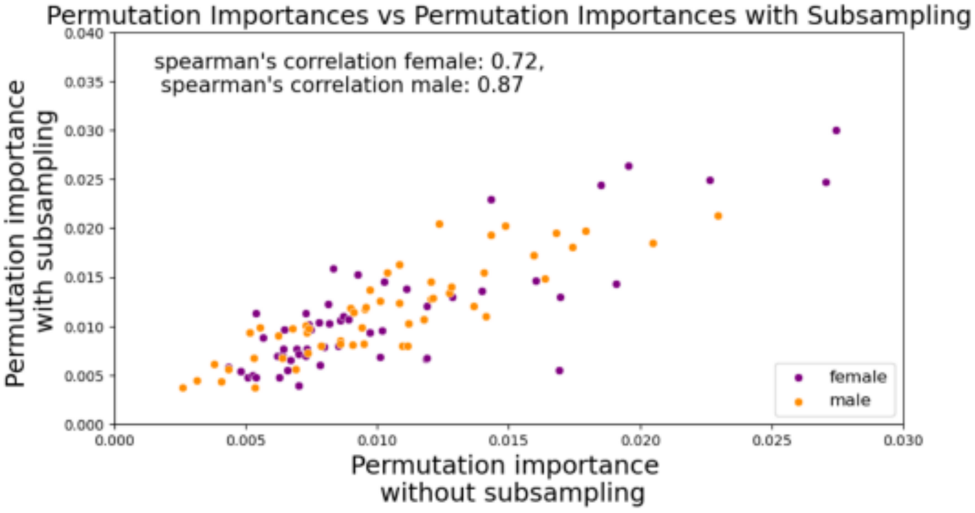

MINOR/TYPO

We have rewritten almost all of these sections and fixed all the minor typos flagged here.

(L. 28-29) "… frequently … often …" seems redundant.

(L. 97) "they do" should be "it does"?

(L. 99) "parameters of was the…" Confusing grammar

(L. 403) "Figure 5S" should be "Figure S5?

Reviewer #3:

This is an interesting MS that offers several contributions, From a behavioral point of view, it demonstrates that ferrets can be trained to categorize and recognize a target word in a stream of other words regardless of its pitch and speaker's voice. This is always a valuable contribution since such tasks can be used in a variety of studies. The other contribution is the utilization of the gradient boosted decision trees to do the analysis, and to demonstrate its power and utility for the analysis of a truly large set of behavioral data.

The paper is very well written and is quite clear and succinct. I do have several questions, suggestions, and perhaps some qualms regarding the interpretations of the results.

1. The ferrets of course were trained to generalize across pitch and voice in all the long initial phases of the training. The animals were trained extensively on the one word "instruments" against any other. The results however are derived presumably starting at the day after they learned the tasks without the pitch shifts. The animals must have improved performance with respect to pitch shifts over time, and did not simply perform at the same good level that is reported. If so, there is no mention of the kinds of errors that they may have made initially before

getting accustomed to the pitch shifts. Is it feasible to do an analysis of that set of initial data separately?

Unfortunately, while this is a fascinating question, in this task - as in almost all of the more complex tasks we train in the lab, when the rules of the task are changed the animals tend to briefly fall apart (as indexed by the number of trials that they do, and their performance on the control trials that they were previously highly competent in). The data we include here is from the point at which those two metrics recover (usually a few days), and from that point performance is stable (but the animals have been exposed to at least a few 100 trials). We have only really managed to gain insight into these early trials in tasks where we have introduced them as probe trials at extremely low probabilities (<10%, e.g. Town and Bizley, J. Neuroscience, 2022). Due to this project being significantly delayed by covid shutdowns etc and our primary aim being to collect a full behavioural dataset and then move on to recording neural activity, prioritizing getting animals to the point we could implant them with recording electrodes as quickly as possible. We agree that in future introducing trials in this manner to understand the animals' innate response would be an interesting experiment.

If we take the first three week's of performance from the point of introducing trials with F0 roving (including all trials, i.e. the initial wobble) the performance looks very similar (data below). We have not included this in the manuscript as it doesn't seem to neatly fit, although we could add it to the supplemental if the reviewers feel there is value to that.

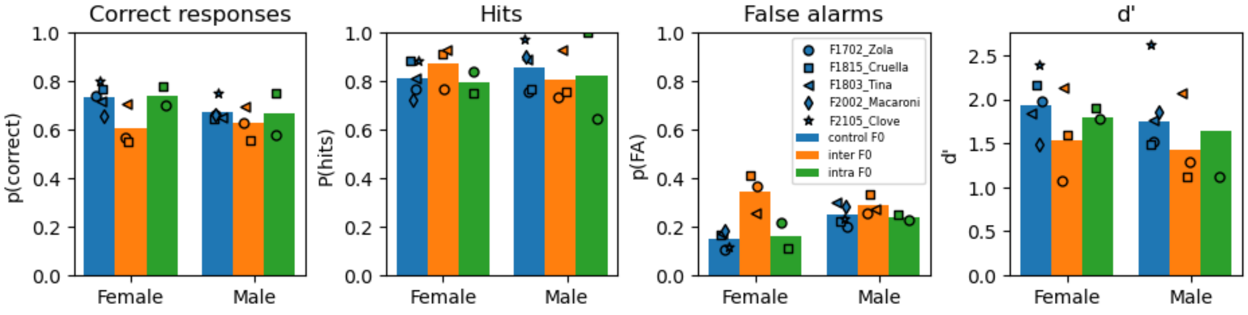

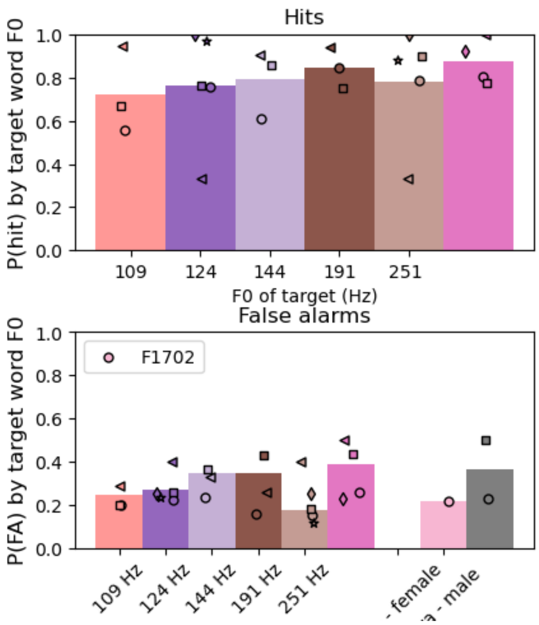

2. It is argues in many places that the reaction time results demonstrate the effects of pitch roving, and hence the value of pitch as a streaming cue. I agree, but not fully for 2 reasons:

(a) Streaming often refers to the existence of sequences from two or more sources. Here, as the authors correctly point ut in the discussion, there was no competing stream of words at all. So the shifts in pitch may have simply caused an uncertainty of the target word because changing any aspect of the target word (pitch, talker, location) is likely to create a variation (within the category of "instruments") which would make it a little more difficult to recognize. Introducing variability after all does affect recognition, but I would not call this affecting the streaming per se.

We agree that there are alternative explanations that cannot be ruled out, and have now elaborated further on these issues in the discussion. We also looked more closely at the effects of timing within a trial and both false alarms and reactions times are higher / slower for early targets, which is at least not inconsistent with the interpretation of streaming. [line 443-450]

The impact of pitch roving in increasing the likelihood of false alarms and slowing reaction times is consistent with ferrets using common pitch to link together sounds over time, offering an advantage for subsequent word recognition. Nonetheless, in the absence of a competing stream of information, we cannot be sure that it is streaming per se or simply that greater changes from word token to token make it a more difficult task. One feature of streaming is that it builds up over time (Moore and Gockel 2012), and consistent with streaming occurring, the likelihood of missing a target was higher, and reaction times were significantly longer for trials in which the target was early in the stream compared to those in which it was in the middle or late in the

stream. We predict that the impact of removing pitch constancy might be more strongly evident in tasks that require separating competing streams.

(b) It would have been nice to rove also the speakers' ID to see how the effect of voice variability affects the recognition. As I mentioned above, just about any variability is likely to affect the target word and the animals would have to suppress that to still recognize the word.

We agree this would have been interesting, and is potentially something we could explore in a followup study.

(c) Streaming formation is a slow process, and so it takes some time for a stream to form. So if streaming specifically played a critical critical, one could show that by finding that the target word was more likely to be correctly recognized after a stream is formed rather than near the beginning. As far as I could see from the results, the animals exhibited the opposite trends, perhaps of impatience!

As the reviewer correctly summises there are many 'nuisance' variables that play into the ferrets behaviour that make it hard to test hypothesis such as these. However, there are hints of what the reviewer suspects in the data though (as noted above, and more easily visible now that we plot the main model effects using the SHAPs scores) - very early in the trial false alarms are more common, misses are more common and reaction times are slower.

3. One interesting finding was that there were specific words that were more likely to be confused with the target word. There is not much analysis of this despite the fascinating possibility that it can shed light on what aspects of the words the ferrets found confusing with the target. Did the authors measure some kind of a distance between the spectrograms of these words relative to the target? Any other analysis?

We have performed additional analysis as outlined to R1 at the beginning of the response letter. This confirms that spectrotemporal similarity (and not amplitude waveform or word duration) was predictive of false alarms.

4. Shifting the pitch of the speakers without compensating for the formant locations etc must have distorted the voices significantly since female formants tend to be higher due to the shorter vocal tracts. So I wonder if part of the pitch shift difficulties was due to the change in voice (assuming of course that ferrets care about vocal tract lengths!).

While the reviewer is correct that we did not alter the formant frequencies, for these modest pitch shifts the voices did not (anecdotally, to us as human observers) sound in anyway distorted however the reviewer is correct that greater pitch-shifts may well have elicited these issues which is one of the reasons that we did not explore greater shifts in F0.

5. The gradient boosted decision trees analysis demonstrated is a nice contribution to the field. The authors point out that there are other generalized “linear” models that could have been used. I think if possible it would be wonderful to demonstrate the results from such a GLM, and to see if it predicts the same subtle effects, and if it does not, then why not? This would be a powerful demonstration of the utility of these alternative nonlinear methods.

Please see our general comments point #2 above which outlines analysis and figures that address this point.
